# Varied Mechanisms and Models for the Varying Mitochondrial Bottleneck

**DOI:** 10.3389/fcell.2019.00294

**Published:** 2019-11-20

**Authors:** Iain G. Johnston

**Affiliations:** Department of Mathematics, Faculty of Mathematics and Natural Sciences, University of Bergen, Bergen, Norway

**Keywords:** mtDNA, bottleneck, development, inheritance, modeling, uncertainty, heterogeneity

## Abstract

Mitochondrial DNA (mtDNA) molecules exist in populations within cells, and may carry mutations. Different cells within an organism, and organisms within a family, may have different proportions of mutant mtDNA in these cellular populations. This diversity is often thought of as arising from a “genetic bottleneck.” This article surveys approaches to characterize and model the generation of this genetic diversity, aiming to provide an introduction to the range of concepts involved, and to highlight some recent advances in understanding. In particular, differences between the statistical “genetic bottleneck” (mutant proportion spread) and the physical mtDNA bottleneck and other cellular processes are highlighted. Particular attention is paid to the quantitative analysis of the “genetic bottleneck,” estimation of its magnitude from observed data, and inference of its underlying mechanisms. Evidence that the “genetic bottleneck” (mutant proportion spread) varies with age, between individuals and species, and across mtDNA sequences, is described. The interpretation issues that arise from sampling errors, selection, and different quantitative definitions are also discussed.

## 1. Introduction

Mitochondria are vital energy-producing compartments in eukaryotic cells. As a result of their evolutionary history, they retain small genomes (mtDNA) which encode important respiratory machinery. In humans and other species, mtDNA molecules are inherited uniparentally, rarely recombine, and can acquire damaging mutations (Wallace and Chalkia, 2013). As hundreds or thousands of mtDNA molecules exist in the same cell, mutations may be present in some but not all molecules: we refer to the fraction of molecules in a cell with a given mutation as the “mutant proportion”. MtDNA molecules within the same cell can harbor many different genetic variants at low proportions, a situation called microheteroplasmy (Guo et al., 2013). The mutant proportion associated with each single genetic variant is of scientific and translational interest, particularly as some variants (e.g., point mutations) have pathological consequences above a certain “threshold” proportion (Rossignol et al., 2003; Johnston and Burgstaller, 2019).

**Figure 1:**
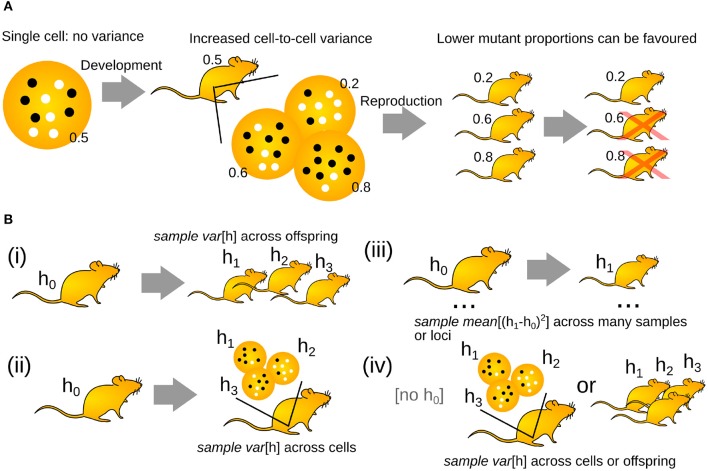
The “genetic bottleneck” increases cell-to-cell mutant proportion spread. **(A)** A mother's life begins as a single cell, with no associated variance in mutant proportion (white circles are wildtype mtDNAs, black circles are mutant mtDNAs; inset numbers give mutant proportion). Development increases cell-to-cell mutant proportion spread in the mother's developing oocytes. In the next generation, oocytes or offspring with lower mutant proportions may be favored. **(B)** Different experimental structures to investigate the generation of mutant proportion spread. (i) Comparing mutant proportion in a mother to her offspring. (ii) Comparing mutant proportion from a reference measurement to a set of individual oocytes. (iii) Comparing mutant proportion differences in a set of mother-child pairs. (iv) Recording mutant proportion differences across oocytes or siblings.

If mothers passed an identical mutant proportion onto each offspring, the buildup of mutations would eventually cause extinction (Muller, 1964). As a result, a developmental process has evolved to generate cell-to-cell variability in mutant proportion in animal germlines (Carling et al., 2011; Jokinen and Battersby, 2013; Stewart and Chinnery, 2015; Zhang et al., 2018)^1^. Thus, while some oocytes may receive higher mutant proportions, some will receive lower loads. Rather than all of a mother's oocytes having 50% mutant proportion, for example, they may range from 20 to 80% (Figure 1A). Oocytes with lower mutant proportions may then go on to become viable offspring, avoiding the buildup of mutation over generations. This increase in the oocyte-to-oocyte variance of mutant proportion is typically discussed as resulting from a “genetic bottleneck.” Increasing cell-to-cell mtDNA variance has also been reported in somatic tissues, suggesting that the “genetic bottleneck” picture may also apply outside the germline (Sekiguchi et al., 2003; Wilton et al., 2018).

Oocyte-to-oocyte, and offspring-to-offspring, variance in mutant proportion is important in the fundamental biology of inheritance, and in human health and disease. While beneficial from an evolutionary perspective, this variance makes it hard to predict mtDNA inheritance patterns. As diseases result from high mutant proportions (Rossignol et al., 2003; Wallace and Chalkia, 2013), this unpredictability makes clinical planning difficult for families carrying dangerous mtDNA mutations (Poulton et al., 1998; Sallevelt et al., 2013). As such, substantial scientific effort is spent characterizing the processes that give rise to mtDNA variability.

The picture of the “genetic bottleneck” can be useful as a simple comparative statistic. However, experimental technology and mathematical theory has now advanced to the stage where we can ask (and begin to resolve) questions about the detailed physical mechanisms behind this genetic behavior. This article will attempt to compare the effective models and detailed mechanisms used to understand this important process, and discuss how these vary through biology and in the scientific literature.

### 1.1. Terminology

The “genetic bottleneck” refers to a genetic quantity—an increase in cell-to-cell variability in mutant proportion. In humans and other animals, the genetic bottleneck is achieved in part (though likely not in full) by a “physical bottleneck” (described further below, and recently reviewed in Zhang et al., 2018). This “physical bottleneck” is a physical reduction in the copy number of mtDNA molecules per cell, which occurs during development. Because the word “bottleneck” appears in both terms, it is sometimes tempting to view the genetic and physical bottlenecks as equivalent. This is not generally the case. Unlike the physical bottleneck, the genetic bottleneck does not directly correspond to a observable number of molecules that can be directly measured by some experiment (Birky, 2001; Johnston and Jones, 2016). A genetic bottleneck of size 10, for example, does not mean that the physical copy number of mtDNAs per cell need ever be 10 at any point during development. As such, the term “mutant proportion spread,” with less physical and more genetic implication, will be used here as a synonym for “genetic bottleneck.” Note that a smaller “bottleneck” leads to more spread and vice versa. As described below, the “genetic bottleneck” (mutant proportion spread) may vary with species, individual, time, mtDNA sequence and other factors. The term “mutant proportion spread” perhaps captures this fluidity more than the more rigid “bottleneck.”

We use “mutant proportion” rather than “heteroplasmy” because a heteroplasmy level over 50% is semantically difficult: the majority mtDNA type should then strictly be considered the reference type, and heteroplasmy redefined with respect to that type.

When taking biological observations and comparing them to models, *population* and *sample* statistics must be considered. Population statistics are summaries of a quantity—like the mean and variance—over the entire population of interest—for example, all oocytes in an organism. Sample measurements of statistics like mean and variance are those derived from a limited number of samples of a larger population. Experimental limitations usually mean that we must consider sample statistics—for example, a set of 20 oocytes from an organism. By contrast, quantitative models typically phrase their predictions in terms of population statistics. Accidents of sampling may lead to differences between sample measurements and population statistics.

When considering these statistics, different studies often use different symbols for the same quantity (Box 1). Here, we will attempt to make equations as verbally “readable” as possible. We write *sample var* [*h*] for sample variances, *sample mean* [*h*] for sample means, *var* [*h*] for population variances and *mean* [*h*] for population means. The sample quantities are computed as described in Box 1.

Box 1 **Calculation and symbols used for mutant proportion statistics.**

Given a set of *n* heteroplasmy measurements *h*_1_, *h*_2_, …, *h*_*n*_, the $sample\;mean\left[h\right]=\frac{1}{n}\overset{n}{\underset{i=1}{\sum }}h_{i}$. Different ways exist to calculate sample variance. Typically, the “unbiased sample variance” is used, that is $s^{2}=\frac{1}{n-1}\overset{n}{\underset{i=1}{\sum }}{\left(h_{i}-sample\;mean\left[h\right]\right)}^{2}$. The “biased sample variance” is the mean squared difference from the mean $s_{n}^{2}=\frac{1}{n}\overset{n}{\underset{i=1}{\sum }}{\left(h_{i}-sample\;mean\left[h\right]\right)}^{2}$. The use of *n* − 1 rather than *n*, known as Bessel's correction, removes bias in the sample variance. As described in the text, studies calculate *sample var* [*h*] using either *s*^2^ (usually for Figure 1Bi,ii,iv) or a mean squared difference approach more like $s_{n}^{2}$ (for Figure 1Biii).

In the literature, sample variances *s*^2^ may also be found represented by *V*(*h*), *V*(*h*), or σ^2^ (but the latter is usually used for population variance). Sample means may be written $\bar{h}$, *E*(*h*), 〈*h*〉, μ (but the latter is usually used for population mean).

## 2. Observations

**Figure 2:**
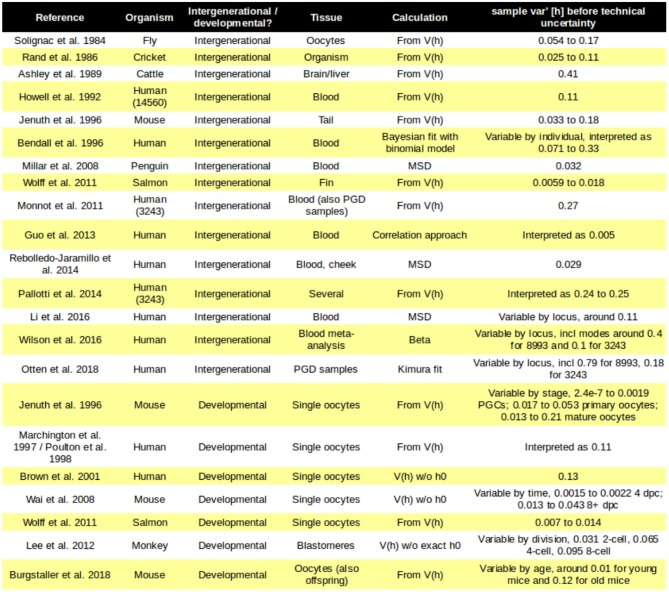
Mutant proportion spreads observed in different systems. Some examples of the diverse mutant proportion spread *sample var′* [*h*] observed experimentally. Loci in brackets refer to specific human mtDNA mutations; PGC, primordial germ cell.

The fundamental observation that implies the existence of a “genetic bottleneck” (mutant proportion spread) is that offspring have different mutant proportions to their parents (Figure 1A). mutant proportions also differ from offspring to offspring. Therefore, at some point(s) between generations, variability in mutant proportion is induced. Parent-to-offspring differences in mtDNA mutant proportion were first reported in cattle (Hauswirth and Laipis, 1982; Ashley et al., 1989; Koehler et al., 1991). Following this, experimental evidence for a “genetic bottleneck” (mutant proportion spread) has been found in animals from flies (Solignac et al., 1984), crickets (Rand and Harrison, 1986), mice (Wai et al., 2008; Burgstaller et al., 2018), salmon (Wolff et al., 2011), and penguins (Millar et al., 2008) to humans (Marchington et al., 1997; Rebolledo-Jaramillo et al., 2014; Li et al., 2016). Some examples of the variety of experimental bottleneck studies are compiled in Figure 2.

A mother starts her life as a single fertilized oocyte. As this is a single cell, there is no cell-to-cell variability in mutant proportion; there is only a single value. The oocytes that later develop in that mother, however, may vary substantially in mutant proportion. This suggests that the reason for offspring differences may be the induction of cell-to-cell mtDNA variability in germline development.

To compute the size of the “genetic bottleneck” (mutant proportion spread), we need a set of “before and after” measurements (Figure 1B). Often, the “before” measurement is taken from a mother. Different studies have different “after” observation structures. In animal models and some human experiments, sets of “after” observations are obtained: for example, measurements across a set of offspring (Figure 1Bi), or a set of single-cell oocyte measurements (Figure 1Bii). Developmental studies aimed at identifying mechanisms rather than “bottleneck size” may take samples of oocytes or their precursors at different stages of development. In other experiments, particularly in human population genetics, a single “after” observation is taken: for example, a single offspring (Figure 1Biii). Many before-after pairs are then used to characterize the population. When a “before” observation is not available, mutant proportion spread may be characterized from “after” measurements and some estimate of the “before” state is constructed (Figure 1Biv). This estimate is often the sample mean of the “after” measurements, thus assuming that no selective shift has occurred.

The mutant proportion variability for a system is typically reported as the sampled variance across a set of “after” observations *sample var* [*h*] (Figure 1B). Most models describing mtDNA statistics (see below) predict that the population variance will follow the form:

(1) $$\begin{array}{l} var\left[h\right]=h_{0}\left(1-h_{0}\right)\times \ldots , \end{array}$$

where … is some expression that may vary according to the model, and *h*_0_ is the mutant proportion in the initial “before” population from which sampling takes place (not the new “after” population). In other words, most models predict cell-to-cell mutant proportion variance to depend on initial mutant proportion *h*_0_, and specifically to be proportional to *h*_0_(1 − *h*_0_).

Because most models have the above form, we often work with a quantity which we here call “mutant proportion spread” but which is usually called “normalized heteroplasmy variance”:

(2) $$\begin{array}{l} {sample\;var}^{\prime }\left[h\right]=\frac{sample\;var\left[h\right]}{h_{0}\left(1-h_{0}\right)}. \end{array}$$

The reason for working with *sample var′* [*h*] is that its normalized value does not typically depend on the specific initial mutant proportion values *h*_0_ from one particular experiment. The results from different experiments, with different values of *h*_0_, can then be more naturally compared.

These variability observations are typically studied from two different perspectives. First, at the “statistical” level: what is the distribution of mutant proportions that will arise from a given mother? This perspective often uses “genetic bottleneck size” as a single number that reflects the observed sample-to-sample mutant proportion spread. Second, at the “mechanistic” level: what physical mechanisms give rise to this distribution of mutant proportions? This perspective attempts to link the coarse-grained outcome of the “genetic bottleneck” to specific, measurable physical rates and properties. In this article, we will first discuss concepts related to this first perspective, before surveying recent progress on the second.

## 3. The “Genetic Bottleneck” Abstracted as Sampling Events or Drift

### 3.1. Abstracting the “Genetic Bottleneck” as a Single Sampling Event

For convenience, studies often describe the “genetic bottleneck” (mutant proportion spread) as the result of a single abrupt event that creates many new individuals, with different mutant proportions, from an initial individual (Figure 3A). In this case, the resulting “bottleneck size” is simply a readout of mutant proportion spread, and does not directly correspond to any physical observable. In particular, it is not generally equal to the minimum copy number of mtDNA molecules (the “physical bottleneck”) (Birky, 2001; Jokinen and Battersby, 2013; Johnston and Jones, 2016; Zhang et al., 2018). This is because the “genetic bottleneck” folds together all mechanisms that can influence mutant proportion spread—the physical bottleneck, cell divisions, random mtDNA dynamics, and so on. The specific number associated with “genetic bottleneck size” may therefore be substantially lower than the physical bottleneck during development.

**Figure 3:**
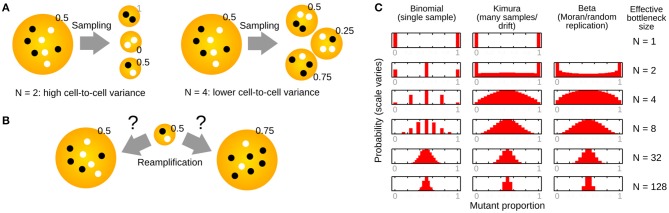
Constructing new populations from random sampling of an initial population. **(A)** Sampling an initial population to construct many new populations of size *N*. Smaller *N* generates more variability between the new populations. **(B)** Reamplification of sampled populations to the original size can be deterministic (left, preserving mutant proportion) or stochastic (right, changing mutant proportion). **(C)** Structures of several distributions related to the study of the “genetic bottleneck” (mutant proportion spread), parameterized by effective “bottleneck size” *N*.

The goal in this perspective is typically to characterize the “genetic bottleneck” (mutant proportion spread) under different conditions. These may involve, for example, different genetic features, different populations, and different species. Knowledge of the value associated with the “genetic bottleneck” (mutant proportion spread) in these cases can inform fundamental biology and clinical planning (Sallevelt et al., 2013).

The concept underlying this approach is a model of “random sampling.” Here, we start with an initial population of mtDNA, with mutant proportion *h*_0_. To create one instance of a final population—for example, the population in one oocyte in the next generation—we randomly sample that initial population. Specifically, we pick at random one member of the initial population and put an mtDNA molecule of that type in our final population. If we are sampling “with replacement,” we retain the picked member in the original population. The alternative is sampling “without replacement,” which involves removing the picked member from the source population so it cannot be picked again.

If we use *N* picks with replacement to construct one new population, and another *N* picks with replacement to construct a second new population, and so on, the new populations will likely differ (Figure 3A). This is because we are likely to choose different numbers of each mtDNA type when we are constructing the new populations.

Quite how different the new populations will be depends on *N*, the number of picks. If we just pick *N* = 1 mtDNA from our source population for each new population, different new populations may differ substantially: each will contain only one mtDNA type, so some populations will have a mutant proportion of 0 and some a mutant proportion of 1. By contrast, if *N* is high, we draw many samples from our initial population, and are likely to end up with new populations that look rather like the initial one (with mutant proportions close to *h*_0_). We can immediately see that our mutant proportion spread (genetic bottleneck) will decrease as *N* decreases (Figure 3A).

This process is called binomial sampling. The actual variance between our new populations is well-known from theory, and is

(3) $$\begin{array}{l} var\left[h\right]=\frac{h_{0}\left(1-h_{0}\right)}{N}. \end{array}$$

A common picture of the “genetic bottleneck” is exactly this *N*. That is, if we observe a certain mutant proportion spread across cells or samples, we work out how large or small *N* would have to be to generate that amount of spread through this binomial sampling, and call this number the “genetic bottleneck.”

How we estimate *N* depends on the structure of our experiment (Figure 1B). First consider the case where we have a single “before” measurement and a set of “after” measurements (for example, a mother mutant proportion and a set of offspring (Figure 1Bi) or oocyte (Figure 1Bii) mutant proportions). Take the sample variance *sample var* [*h*] of the “after” measurements. Call the “before” measurement *h*_0_. Then the definition of “bottleneck size” is often taken to be

(4) $$\begin{array}{l} N=\frac{h_{0}\left(1-h_{0}\right)}{sample\;var\left[h\right]}=\frac{1}{{sample\;var}^{\prime }\left[h\right]} \end{array}$$

based on this binomial sampling picture. If *h*_0_ is not known, as in Figure 1Biv, it is sometimes estimated to be equal to *sample mean* [*h*]. That is, the assumption is made that no shift in mutant proportion has taken place due to selection or accidents of sampling.

The idea here is to convert a less intuitive quantity *sample var′* [*h*] into a more intuitive one (an effective number of segregating units). However, this binomial sampling picture has some issues. First, it does not correspond to a plausible biological mechanism. Development does not involve a single, abrupt sampling event. How reamplification of mtDNA back to its original level takes place is rarely considered (Figure 3B), although models for reamplification do exist (see below). Second, and related, a binomial sampling regime predicts a binomial distribution for final mutant proportion (Figure 3C). For a small value of *N*, this means that mutant proportion can only take one of a restricted set of values. For example, if *N* is 4, we would only expect mutant proportions of 0, 25, 50, 75, and 100% after sampling. Other models have been proposed to address these shortcomings (see below).

Next, consider the case where we have a set of paired “before” and “after” observations (for example, the mother-single offspring pairs in Figure 1Biii). The prevailing approach to calculate a “bottleneck size” here is via an expression derived in references (Millar et al., 2008; Hendy et al., 2009) based on

(5) $$\begin{array}{l} N=\frac{h_{0}\left(1-h_{0}\right)}{sample\;mean\left[{\left(h-h_{0}\right)}^{2}\right]}. \end{array}$$

Here, the spread of “after” measurements $sample\;var\left[h\right]=\frac{1}{n-1}\underset{i}{\sum }{\left(h_{i}-sample\;mean\left[h\right]\right)}^{2}$ has been replaced by the mean square difference between the “before” and “after” measurements $\frac{1}{n}\underset{i}{\sum }{\left(h_{i}-h_{0}\right)}^{2}$. That is, the approach assumes that the average “after” measurement *sample mean* [*h*] is equal to the “before” measurement *h*_0_—in other words, that no shifts in mean mutant proportion act between generations. If selection is in fact present, Equations (4) and (5) give different results, and Equation (5) quickly fails to capture the true bottleneck size even in abstracted systems (Figure 4). Because Equation (5) deals with squared differences, selective shifts in different directions do not “cancel out” but rather reinforce the resultant discrepancy.

**Figure 4:**
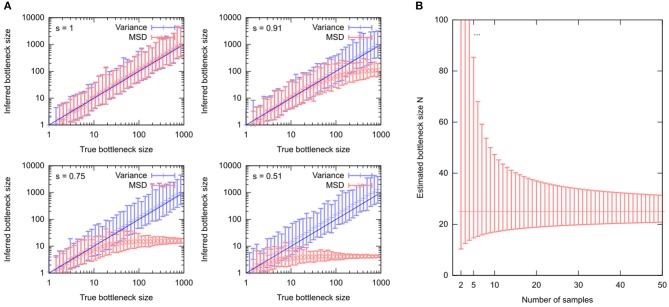
Estimating the “genetic bottleneck” from different data sources. **(A)** Simulated data, comparing estimates of “bottleneck size” *N* using either 16 “after” measurements (“Variance”) or 16 sets of single “after” measurements (“MSD”). Estimates are performed in the presence of different levels of selection (*s* = 1, no selection; decreasing *s* is increasing selective pressure). **(B)** Uncertainty in a “bottleneck size” estimate using *mean* [*h*] = 0.5, *var* [*h*] = 0.01, and different numbers of samples *n*. Particularly for *n* < 10, bottleneck size estimates can have large uncertainty.

### 3.2. The “Genetic Bottleneck” Abstracted as Several Sampling Events or Drift

A single binomial sampling event does not represent a real biological mechanism. To improve this picture, some studies consider the “genetic bottleneck” (mutant proportion spread) as arising from a series of sampling events, modeling cell divisions that randomly partition mtDNA molecules between cells. Early work on mtDNA inheritance (Solignac et al., 1984; Rand and Harrison, 1986; Ashley et al., 1989; Howell et al., 1992) drew on a classical result from Sewall Wright (Wright, 1942, 1984) to this end. This result describes the spread of allele frequencies due to “accidents of sampling” in repeated generations, where the individuals in one generation are a random sample from the previous generation. For mtDNA, this “Wright equation” predicts

(6) $$\begin{array}{l} var\left[h\right]=h_{0}\left(1-h_{0}\right)\left(1-{\left(1-\frac{1}{N}\right)}^{kn}\right), \end{array}$$

where *k* is the number of random samplings per generation (for example, the number of cell divisions in germ line formation) and *n* is the number of generations. The reader will notice that if *kn* = 1, describing a single sampling event as above, Equation (4) is recovered.

For convenience, some studies have since defined new “bottleneck parameters” to simplify this expression. One choice is to set α = (1 − 1/*N*)^*k*^. Another more recent alternative is to define *b* = exp(−*g*/*N*). Here *g* = *kn* represents an amalgamated number of samplings, and the exponential form is used for algebraic convenience because exp(−*g*/*N*) ≃ (1−1/*N*)^*g*^. In these cases, Equation 6 becomes:

(7) $$\begin{array}{l} var\left[h\right]=h_{0}\left(1-h_{0}\right)\left(1-\alpha^{n}\right)≃h_{0}\left(1-h_{0}\right)\left(1-b\right). \end{array}$$

The advantage of using these “bottleneck parameters” is that they fold together two unknown quantities: the number of generations and the effective population size. Under Equation (6), readouts of “bottleneck size” (mutant proportion spread) using *N* are contingent on a particular choice of *g*, the number of generations for which the bottleneck applies (Figure 5A). Readouts using *b* (and α) absorb this dependency, providing a simple readout of mutant proportion spread that makes no assumptions about the number of sampling events (Figure 5B). Given a number of generations, *N* can be recovered from *b* via *N* = −*g*/ln *b*.

**Figure 5:**
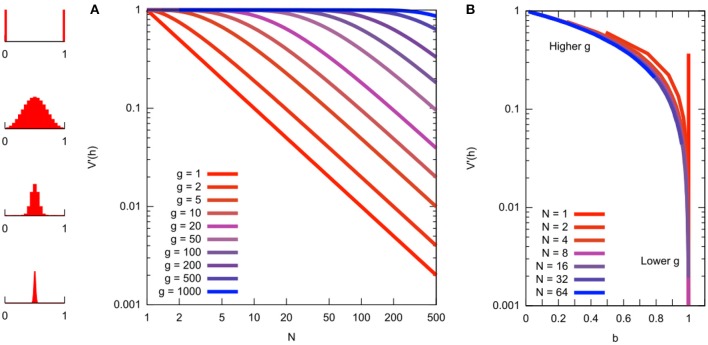
Relationship between different quantities related to mutant proportion spread. **(A)** Mutant proportion spread with different sampling parameters. Horizontal axis gives “bottleneck size” *N*. Vertical axis gives corresponding mutant proportion spread *sample var′* [*h*] (from Equation 6 with *g* = *kn*). Different traces are for different numbers of sampling events *g*. To convert a *sample var′* [*h*] value to a “bottleneck size” *N*, choose the number of sampling events *g* and read off the corresponding value (distribution sketches at the far left give illustrations of the Kimura distribution for the various *sample var′* [*h*] values). **(B)** Mutant proportion spread with summary parameter *b*, from simulated sampling dynamics. Horizontal axes gives “bottleneck parameter” *b*. Vertical axis gives corresponding mutant proportion spread *sample var′* [*h*]. Behavior at different *N* and *g* are now “folded together,” collapsing on the same line.

An approximation of the cell-to-cell distribution of mutant proportion under these repeated-sampling models is the so-called Kimura distribution (Wonnapinij et al., 2008) (Figure 3C). Strictly, the assumptions involved in deriving this approximation rely on *N* being large (Kimura, 1955). However, the Kimura distribution reproduces intuitive behavior for the distribution of mutant proportion under drift, and several studies use a fit to the Kimura distribution to estimate *b* (Wonnapinij et al., 2008; Otten et al., 2018).

The relationship between these quantities *N*, *b*, *g*, and *sample var′* [*h*] is illustrated in Figure 5, which may serve as a reference for comparison of reported “genetic bottleneck” (mutant proportion spread) statistics in different studies.

### 3.3. Drift Manifest Through Random Replication

One issue with a simple sampling picture is that it predicts a set of cellular mtDNA populations consisting of *N* molecules. In most circumstances, this *N* value is much lower than the size of typical cellular populations. For example, in animal germline development, the number of mtDNA molecules per cell is amplified several orders of magnitude from a minimum copy number back to a functional level (Cree et al., 2008; Wai et al., 2008; Cotterill et al., 2013; Zhang et al., 2018). If this reamplification happened perfectly deterministically, no further change in *var* [*h*] would occur. However, cell biology is rarely deterministic, and there is good reason to believe that this reamplification process involves a random component (Birky, 1994; Chinnery and Samuels, 1999; Capps et al., 2003; Johnston et al., 2015).

Several recent studies have considered models for this reamplification (Johnston and Jones, 2015; Wilson et al., 2016). Most are based on the idea of random mtDNA replication. That is, an mtDNA molecule is randomly chosen from the current population, and replicated. Then a new mtDNA molecule is randomly chosen and replicated, and so on until the desired population size is achieved. This is a modified Moran model (Moran, 1958) (the usual Moran model involves removing one molecule per replication, so that overall population size remains constant), also known as a Pólya urn model (Eggenberger and Pólya, 1923; Johnson and Kotz, 1977).

In the limit of infinite reamplification, the model gives rise to a beta distribution for mutant proportion spread (Figure 3C). Infinite reamplification may not seem realistic, but actually the structure of this distribution is quickly stabilized after a relatively small number of replications, so the simple infinite limit is similar to more reasonable cases. However, results also exist for intermediate cases, and their exploration may be a fruitful area of future research. The beta distribution takes two parameters, α and β, intuitively corresponding to the number of mutant and wildtype molecules in the cell before any replication. If we α = *h*_0_*N* and β = (1 − *h*_0_)*N*, the mean of the beta distribution is *mean* [*h*] = *h*_0_ as expected, and the variance of the beta distribution is

(8) $$\begin{array}{l} var\left[h\right]=\frac{h_{0}\left(1-h_{0}\right)}{N+1}. \end{array}$$

### 3.4. Uncertainty

The “genetic bottleneck” (mutant proportion spread) is a readout of variance. Revealing trends in cell-to-cell variance is more challenging than revealing trends in average behavior, and requires more data. Wonnapinij et al. (2010) have drawn attention to the challenging nature of obtaining reliable estimates of mutant proportion spread. Uncertainty in estimated mutant proportion spread is often large, challenging precise estimates of the “bottleneck size” and leading to variability in these estimates. Even in the case of no technical error (see below), sampling errors can lead to large variability in estimates of “bottleneck size,” particularly if fewer than 10 samples are used (Figure 4B).

Uncertainty in readouts of variance can be an unintuitive quantity. We are perhaps more used to thinking about mean values as the quantity of interest, with variance around a mean value corresponding to uncertainty. However, we can—and should—also describe and estimate the uncertainty associated with an observation of variance.

One way to estimate uncertainty in *sample var* [*h*] involves assuming that mutant proportion samples are drawn from a normal distribution. This is not generally the case (as seen in Figure 3C), but is a simple illustration that may be applied when spread is low. Confusingly, there are two expressions in circulation for the sampling error in this case. Which of these values gets used depends on how the variance was computed. If the variance is calculated using an estimate of the mean taken from the same dataset (employing Bessel's correction, as with many “after” measurements), Wonnapinij et al. (2010) cite:

(9) $$\begin{array}{l} SE\left[sample\;var\left[h\right]\right]=var\left[h\right]\times \sqrt{\frac{2}{n-1}}, \end{array}$$

for the standard error in *sample var* [*h*], where *n* is the number of samples used to characterize *sample var* [*h*]. If the mean is estimated from a different source (omitting Bessel's correction, as with mean-squared-difference calculations using a single “after” measurement), an estimate of the variance of the sample variance is (*var* [*h*])^2^(*n* − 1)/*n*^2^, as quoted in reference (Millar et al., 2008), corresponding to a standard error of $var\left[h\right]\times \sqrt{\left(n-1\right)/n^{2}}$. The standard error associated with a variance measurement can then be estimated by using *var* [*h*] ≃ *sample var* [*h*] in these expressions.

However, for wide spreads or means close to 0 or 1, mutant proportion distributions do not have normal structure. In this case (Wonnapinij et al., 2010), cite a more general result:

(10) $$\begin{array}{l} \text{SE}\left[sample\;var\left[h\right]\right]=\sqrt{\frac{1}{n}\left(D_{4}-{\left(var\left[h\right]\right)}^{2}\times \left(\frac{n-3}{n-1}\right)\right)} \end{array}$$

where $D_{4}=\left(n-1\right)/n^{3}\times \left(\left(n^{2}-3n+3\right)\mu_{4}+3\left(2n-3\right)\mu_{2}^{2}\right)$, and $\mu_{2}=1/n\overset{n}{\underset{i=1}{\sum }}{\left(h_{i}-h_{0}\right)}^{2}$, $\mu_{4}=1/n\overset{n}{\underset{i=1}{\sum }}{\left(h_{i}-h_{0}\right)}^{4}$. While more complicated in structure, all these quantities can readily be worked out from the set of observed mutant proportion measurements.

All these expressions have the standard error of *sample var* [*h*] scale roughly with the observed value divided by $\sqrt{n}$. Thus, unless a large number *n* of samples are used to characterize mutant proportion spread, the associated uncertainty in *sample var* [*h*] can be rather high. As “bottleneck size” estimates depend on 1/*sample var* [*h*], the corresponding uncertainty can be enormous for low sample sizes (Figure 4B).

These expressions are based on the statistics of sampled variances, and assume that the sample mutant proportion values themselves have no associated uncertainty (in other words, there is no technical error associated with the genetic measurement). Technical error should also be included in the uncertainty associated with these estimates. Several studies include considerations of technical error in their estimates of mtDNA statistics (Bendall et al., 1996; Millar et al., 2008; Li et al., 2016; Wilson et al., 2016). This is typically achieved through simple uncertainty propagation, that is, considering an observed variance to be a combination of natural variance and technical variance. The technical variance may either be quantified through experimental calibration (Millar et al., 2008) or as part of a statistical inference process (Bendall et al., 1996; Li et al., 2016; Wilson et al., 2016).

### 3.5. Results

Early reports of the size of the genetic bottleneck (mutant proportion spread) varied substantially across organisms. Contributing to this variability was the fact that different studies used different values of *kn* in Equation (6). These different values reflected, for example, estimates of the number of cell divisions involved in germline development in different species. More recently, it has become more common to set *kn* = 1 and assume a single binomial sampling event, or to use a “bottleneck parameter,” usually *b*, to summarize mutant proportion spread as above. Figure 2 summarizes the mutant proportion spreads observed in several key experimental studies across species.

The rapid intergenerational shifts observed in cattle (Hauswirth and Laipis, 1982; Koehler et al., 1991) have given rise to the highest mutant proportion spread values so far observed. Insects appear to have lower mutant proportion spreads (Solignac et al., 1984; Rand and Harrison, 1986). In mice, several experiments have observed the increase of mutant proportion spread through germline development (Jenuth et al., 1996; Wai et al., 2008). Fish show similar behavior (Wolff et al., 2011).

Mutant proportion spread in humans was observed some time ago (Bendall et al., 1996; Marchington et al., 1997), but its magnitude remains debated. Variability in the behavior of mutant proportion spread was quickly apparent. Blok et al. found dramatic skew toward extreme mutant proportions in transmission of the 8993 mutation (Blok et al., 1997). Lutz et al. (2000) found evidence for variable mutant proportion spread in a human family; while they did not provide quantitative estimates they noted that the different spreads they observed suggest a varying “bottleneck size” which could be very small. Bendall et al. (1996) used a Bayesian approach to show that it was unlikely that their study families had the same “bottleneck size.” More recently, two large-scale population-genetic studies suggest rather different “bottleneck sizes” (Rebolledo-Jaramillo et al., 2014; Li et al., 2016). Pathogenic mutations seem to involve more mutant proportion spread, particularly the 8993 mutation (Blok et al., 1997; Monnot et al., 2011; Wilson et al., 2016; Otten et al., 2018). Ongoing preimplantation genetic diagnoses approaches continue to provide data on mutant proportion spread at different developmental stages (Monnot et al., 2011; Treff et al., 2012; Sallevelt et al., 2013). Pallotti et al. (2014) performed a meta-analysis of 3243 bottlenecks along with their own experiments and found reasonable consistency in mutant proportion spread. Notably, different studies still use different protocols for reporting a “bottleneck size,” sometimes setting *g*(= *kn*) = 24 or *g*(= *kn*) = 1 in Equation (6).

While not a focus of this article, we note that genetic bottlenecks (increasing mutant proportion spread) (Sekiguchi et al., 2003; Wilton et al., 2018) and physical bottlenecks (Cao et al., 2007; Otten et al., 2016; Floros et al., 2018) have also been reported in somatic tissues.

## 4. The “Genetic Bottleneck” as a Set of Physical Processes

In parallel with statistical characterization of the “genetic bottleneck” (mutant proportion spread), related research attempts to understand the physical processes that give rise to an observed “genetic bottleneck” (mutant proportion spread) in a given system. The goal here is typically to identify biological mechanisms and potential targets for intervention.

**Figure 6:**
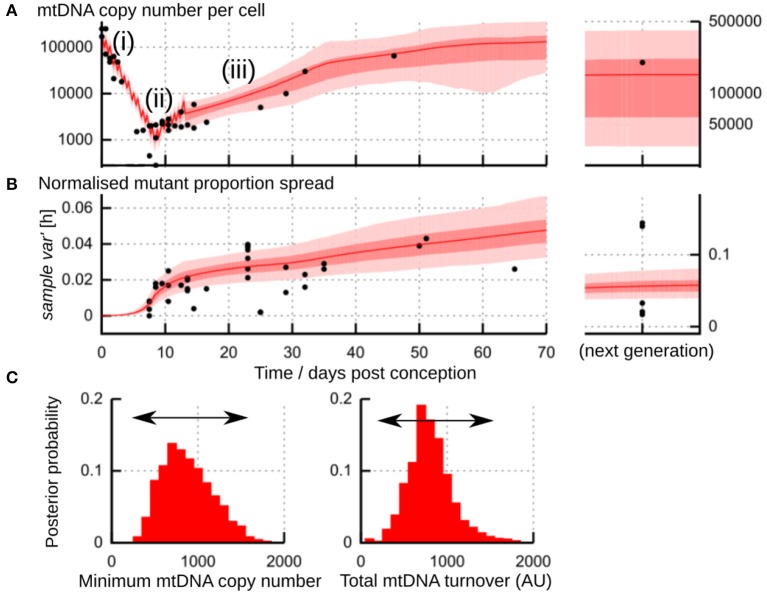
Flexibility in the physical processes underlying the “genetic bottleneck.” **(A)** Physical mtDNA dynamics (copy number per cell) in mouse germline development. (i) repeated cell divisions after fertilization with little compensatory mtDNA replication lead to a drop in copy number to a minimum “physical bottleneck” (ii). Copy number is subsequently reamplified (iii) through later development. **(B)** Dynamics of *sample var′* [*h*] in mouse germline development. In **(A,B)**, datapoints (black) are amalgamated from references (Jenuth et al., 1996; Cao et al., 2007; Cree et al., 2008; Wai et al., 2008); shading shows posterior distributions from the most-supported “birth-death-partitioning” model, involving random mtDNA turnover and partitioning at cell divisions (Johnston et al., 2015). **(C)** Observations of physical and genetic dynamics **(A,B)** are best fit by a model that allows a flexible “physical bottleneck” (left, posterior distribution) which can be compensated by a flexible amount of mtDNA turnover (right, posterior distribution). Figure uses results from Johnston et al. (2015).

A plausible physical mechanism for the “genetic bottleneck” (mutant proportion spread) must account for both physical and genetic observations over time during development. The physical observations involve mtDNA copy number per cell and the occurrence of cell divisions; the genetic observations involve cell-to-cell variability in mutant proportion. An example from a meta-analysis of mouse observations is shown in Figures 6A,B. The joint prediction of these physical and genetic observations is very important because it constrains the mechanisms that are possible—for example, the size of the physical bottleneck, the timing of cell divisions, and the rate of reamplification all influence the resulting genetic statistics of mtDNA populations.

While not a focus of this article, the specific genetic players behind the physical processes below are increasingly being revealed, and have been reviewed in, for example, references (Carling et al., 2011; Jokinen and Battersby, 2013).

### 4.1. The Physical Bottleneck During Development

One process that occurs during germline development in animals is a physical reduction in the number of mtDNA molecules per cell (Zhang et al., 2018). This reduction is observed in animals including mice (Cao et al., 2007; Cree et al., 2008; Wai et al., 2008), fish (Wolff et al., 2011; Otten et al., 2016), sheep (Cotterill et al., 2013), and humans (Floros et al., 2018). For some time after fertilization, cell divisions repeatedly halve the cellular mtDNA population, with little compensatory replication. This halving leads to a pronounced drop in mtDNA copy number per cell (Figure 6Ai). A fertilized oocyte typically contains many mtDNA molecules [hundreds of thousands in mice (Cree et al., 2008; Wai et al., 2008); around a million in humans (Floros et al., 2018)]. The size of the physical bottleneck—that is, the lowest copy number of mtDNA per cell during development—remains debated, but is often orders of magnitude lower; Zhang et al. (2018) have recently provided a survey of mtDNA reduction in different species. In mice, the lowest copy number may lie between 200 and 1,000 (Cao et al., 2007, 2009; Cree et al., 2008; Wai et al., 2008; Johnston et al., 2015) (Figure 6Aii). In humans, mean copy numbers around 1400 are observed in progenitor germ cells (Floros et al., 2018). In zebrafish, decreases from tens of millions to hundreds of mtDNAs per cell are observed (Otten et al., 2016). The copy number of mtDNA during development seems to depend on genetic characteristics of the mtDNA (Monnot et al., 2013), potentially making the physical bottleneck sequence-dependent.

Pictured as drawing a random selection of mtDNA molecules from a larger population, copy number reduction provides a way to generate variability between cells. Additionally, the magnitude of variability generated through other random sampling processes is amplified by low copy numbers.

### 4.2. Random Replication of a Subset of mtDNA Molecules

In this mechanism, at some point(s) in germline development, a random subset of a cell's mtDNA population is allowed to replicate, while all others are eventually subject to degradation or loss (Wai et al., 2008). This subset may be, for example, those mtDNAs within a certain distance of the nucleus (Wallace, 2018). As the random subset chosen will differ in different cells, this process imposes a natural sampling inducing variance between cells. A smaller subset of molecules will lead to more mutant proportion spread.

Wai et al. observed a sharp increase in mutant proportion spread in mice aged between 4 and 8 days (as in Figure 6B) (Wai et al., 2008; Samuels et al., 2010). Using microscopy, they showed that only a subset of mtDNA molecules was involved in replication at a given time. They propose this subset replication model as the mechanism by which variability is generated at this development stage (folliculogenesis, Figure 6Aiii). Johnston et al. (2015) suggest that this observation is also compatible with random mtDNA turnover (see below), where a non-fixed subset of mtDNAs is expected to be involved in replication at any given time.

The random replication model described above, connected to the beta distribution, can describe the dynamics of the subset-replication model. Care must be taken here to ensure that physical copy number dynamics are reproduced: for example, the small amount of replicating mtDNAs must balance the large number of degrading mtDNAs as copy number is amplified (Figure 6Aiii).

### 4.3. Random Partitioning of mtDNA Molecules at Cell Divisions

This mechanism is possible during specific times when cells are undergoing divisions. At division, a “parent” cell distributes its population of mtDNA to its two “daughter” cells. The assignment of each mtDNA molecule to one or the other daughter may follow a random process (Birky, 2001; Huh and Paulsson, 2011; Johnston et al., 2012). In this case, each division will increase the cell-to-cell mutant proportion variability between daughter cells. If mtDNA molecules are partitioned in clusters, this increase will be faster (Cao et al., 2007). Larger clusters will lead to more mutant proportion spread.

Whether the “unit of inheritance” of mtDNA is a single molecule or a cluster is a debated question. MtDNA within mitochondria is packaged into complexes called nucleoids. These were thought to contain around 5–10 mtDNA genomes (Jacobs et al., 2000; Cao et al., 2007; Khrapko, 2008), suggesting that clusters of mtDNA may be the natural state. However, evidence from microscopy suggests that nucleoids may only contain around 1 mtDNA genome (Kukat et al., 2011). Model selection for mouse germline development (Johnston et al., 2015) and human transmission (Li et al., 2016) both suggest that single mtDNA molecules are the unit of inheritance.

Observations in rhesus monkeys (Lee et al., 2012) showed a dramatic induction of variance by the 8-cell stage, presumably due to random partitioning of mtDNAs over the first three cell divisions. In this study, mtDNA admixtures in oocytes were created by fusing two cytoplasm halves from different oocytes. Cell divisions then immediately followed the construction of these admixed oocytes. It is thus not inconceivable that physical heterogeneity in the distribution of mtDNA molecules, remaining from the cytoplasm fusion, may contribute to this high mutant proportion spread. For example, if the fusion process created a “north hemisphere” containing exclusively one mtDNA type and a “south hemisphere” containing exclusively the other, and the first cell division occurred along the “equator,” the resultant cells would then immediately have maximum mutant proportion differences. Natural systems may be expected to have more physically mixed mtDNA populations, and so potentially show less extreme mutant proportion spreads in these early stages.

The “repeated sampling” approaches above attempt to model cell divisions during development as a series of random binomial samples. Partitioning dynamics can also be embedded in stochastic models of mtDNA replication and degradation (Johnston and Jones, 2015, 2016). Much of this work assumes binomial partitioning; however, recent work in yeast has suggested that partitioning of mtDNA is tighter than binomial sampling (Jajoo et al., 2016). Mathematical results do exist for more controlled partitioning, or the partitioning of clusters of mtDNA (Johnston and Jones, 2015) but are often complicated, so simulation is often used to make quantitative predictions in these cases (Johnston et al., 2015; Li et al., 2016).

### 4.4. Random Turnover of mtDNA Molecules

MtDNA replicates and degrades quasi-independently of the cell cycle. The noisy environment of the cell means that these processes have a random component (Birky, 1994; Chinnery and Samuels, 1999; Capps et al., 2003; Johnston et al., 2015). The ongoing action of this random turnover creates cell-to-cell mutant proportion variability. For example, two cells that start with identical mtDNA populations will diverge over time, as different molecules undergo replication and degradation. Faster turnover, or turnover of clusters, will lead to more mutant proportion spread.

To account for the full set of processes that an individual mtDNA molecule may undergo, several stochastic modeling approaches have been developed (reviewed in Hoitzing et al., 2017). These approaches model every individual mtDNA molecule in a cell and subjects them to the physical processes that we may expect to occur during development. Typically, these processes will have a random component, so that if the model is simulated twice, the precise outcomes will differ. These differences can be used to characterize the variability supported by different mechanisms.

A well-known model involves “relaxed replication,” that is, replication of mtDNA independent of the cell cycle (Birky, 1994). Models of this process typically involve mtDNA molecules degrading with a fixed rate, and replicating randomly with a rate that depends on population size (Chinnery and Samuels, 1999; Capps et al., 2003). This model generates variability over time because of these random dynamics. Cree et al. propose this mechanism, amplified by the physical bottleneck, to generate mutant proportion spread in mouse development (Cree et al., 2008).

More recently, the different ways that the cell could control this replication rate have recently been explored in detail using “birth-death” models (Johnston et al., 2015; Johnston and Jones, 2016; Hoitzing et al., 2019). Strikingly, this work showed that no matter how the cell controls mtDNA replication, if there is some mutant proportion, the variance of this mutant proportion will increase linearly over time.

Specifically, in a population of *N* mtDNAs, random turnover of molecules with rate β over time *t* gives rise to the behavior

(11) $$\begin{array}{l} {sample\;var}^{\prime }\left[h\right]=\frac{2f\beta t}{N}, \end{array}$$

so that, for example, a year of mtDNA turnover, with average rate one degradation event per week, in a cell with 1,000 mtDNA molecules would give a mutant proportion spread of (2 × 52)/1, 000 = 0.104. This would be interpreted as a “bottleneck size” around 9.6. In followup theoretical developments (Aryaman et al., 2019), the factor *f* in Equation (11) has been shown to be the fraction of unfused mitochondria, that is, mitochondria containing mtDNAs subject to mitophagy (Youle and Narendra, 2011; Diot et al., 2016). Mitochondrial quality control, linked to fission-fusion dynamics, contributes to the turnover of mitochondria in the cell (Twig et al., 2008) and provides one way that mitochondrial dynamics may influence both mean and variance dynamics of mtDNA populations (Hoitzing et al., 2015; Johnston, 2018; Latorre-Pellicer et al., 2019). Higher rates of quality control related turnover can result in higher cell-to-cell mutant proportion variance (Johnston et al., 2015) [and, if mitochondria associated with one mtDNA type are preferentially degraded, this selective pressure will also influence mean mutant proportions (Twig et al., 2008; Hoitzing et al., 2015)]. Equation (11) provides a coupling between the physical fission-fusion dynamics of mitochondria and the time behavior of mtDNA mutant proportion spread (Hoitzing et al., 2015; Johnston, 2018; Aryaman et al., 2019).

### 4.5. Combinations of Mechanisms

Several of these processes are conceptually linked. For example, when a cell divides, it loses around half of its mtDNA content, immediately restricting the subset of mtDNAs that are available for replication. If mtDNA molecules are involved in ongoing random turnover, only a subset of molecules will be replicating at any given time (Johnston et al., 2015).

In each of these cases, a smaller mtDNA population acts to amplify increases in mutant load spread, because the influence of random events is less “smoothed out” in small populations. Therefore, we can end up with the same amount of spread by either (i) generating a smaller amount and amplifying it more through small population size; or (ii) generating a larger amount and amplifying it less. Indeed, analysis of mouse data suggests that the same amount of spread can be achieved with a small physical bottleneck and less mtDNA turnover (less generation, more amplification) or a large physical bottleneck and more mtDNA turnover (more generation, less amplification) (Johnston et al., 2015) (Figure 6C). This flexibility may help reconcile differing reports on the size of the physical bottleneck (Cao et al., 2007, 2009; Cree et al., 2008; Johnston et al., 2015). It is not inconceivable that some mechanism may allow the cell to sense and control this choice, so that, for example, embryos with slightly lower mtDNA turnover have their mtDNA populations depleted more to compensate.

To consider these mechanisms together, the birth-death framework above was coupled to a description of cell divisions to provide a detailed stochastic model of germline development in mice (Johnston and Jones, 2015; Johnston et al., 2015). Compared to other detailed models, this birth-death-partitioning model provided the best fit to a meta-analysis of existing physical and genetic data. The best model for cell-to-cell spread of mutant proportion had two components: a contribution from partitioning at cell divisions and a contribution from ongoing drift due to mtDNA turnover.

The birth-death-partitioning model provides closed-form, though complicated, expressions for full distributional details of mutant proportion at all times through development, which well-predicted independent experimental observations of mutant proportion distributions in oocytes (Johnston et al., 2015). The combined birth-death-partitioning model was also used to provide an update to the Wright equation (Equation 6) to include random mtDNA turnover (Johnston and Jones, 2016), predicting:

(12) $$\begin{array}{l} {sample\;var}^{\prime }\left[h\right]=1-{\left(1-\frac{1}{N}\right)}^{g}+\frac{4t}{3N\tau }, \end{array}$$

where *N* is now a physical mtDNA copy number, *g* a physical number of cell divisions, *t* is time and τ is the timescale of mtDNA degradation. Append:

The final term in Equation (12) estimates the ongoing increase in mutant load spread due to mtDNA turnover, increasingly linearly with time *t*.

## 5. Recent Topics

### 5.1. Model Selection and Predictions

We have discussed a range of different proposed mechanisms for the “genetic bottleneck” (mutant proportion spread). A comparatively recent set of studies has attempted to identify the mechanisms that are most supported by data. This has been attempted through the use of model selection (Kirk et al., 2013), a process that compares the statistical support for different mechanisms while guarding against overfitting. Li et al. used likelihood-based model selection with a human dataset to provide support for a “genetic bottleneck” (mutant proportion spread) that varies for different sequences and involves individual mtDNAs (rather than clusters) as segregating units (Li et al., 2016). Johnston et al. used likelihood-free model selection for mouse data to identify the mechanism(s) most supported by data. They found little support for partitioning of clustered mtDNA, and most support for the birth-death-partitioning model above, which was further supported by followup experiments (Johnston et al., 2015). A theoretical comparison of different models for mtDNA control (Johnston and Jones, 2016) revealed the above principles of increasing variance that hold regardless of which specific mechanism is true. More recently, large-scale inter-generational data from mice was used in a statistical framework to identify which processes influence mtDNA statistics during development and aging (Burgstaller et al., 2018).

These detailed mathematical models present the opportunity to refine the prediction of mutant proportion distributions. The birth-death-partitioning model predicted distributional details of oocyte mutant proportion in developing mice (Johnston et al., 2015). Based on the picture of increasing mutant proportion spread in aging oocytes, a simple model involving a variation of a logit-normal distribution for mutant proportion predicted distributional details of mutant proportion in mouse litters (Burgstaller et al., 2018).

### 5.2. Sequence-Specific Behavior in Mutant Proportion Spread

Substantial recent attention has been focussed on whether the genetic bottleneck (mutant proportion spread) is sequence-specific. Evidence for this hypothesis includes observations from different pathological mtDNA mutations (Monnot et al., 2011; Wilson et al., 2016; Otten et al., 2018). Consideration of different human variants in a population genetic context also suggests that the magnitude of the genetic bottleneck (mutant proportion spread) depends on the specific variant under investigation (Li et al., 2016). A particularly striking difference appears to exist between the 3243 and 8993 mutations (Monnot et al., 2011; Wilson et al., 2016; Otten et al., 2018). The aforementioned population study (Li et al., 2016) also found a variable-size bottleneck to be most statistically supported for non-pathological mutations.

As discussed throughout, sequence-specific proliferative advantages of one mtDNA type over another can confound attempts to analyse the genetic bottleneck (mutant proportion spread). A sequence-specific increase in mutant proportion spread can arise without a proliferative difference between sequences: for example, if one sequence experiences both higher replication and degradation rates, increasing random turnover without an overall selective advantage. Conversely, under some experimental designs, sequence-specific differences in the behavior of mean mutant proportion (i.e., proliferative differences) could be interpreted instead as differences in mutant proportion variance if it is assumed that no proliferative differences exist (as in Figure 4A). Further theoretical work unpicking the behavior of mtDNA statistics as mean and variance change together will be useful in interpreting these observations.

### 5.3. Ongoing Increase of Mutant Proportion Spread During Aging

**Figure 7:**
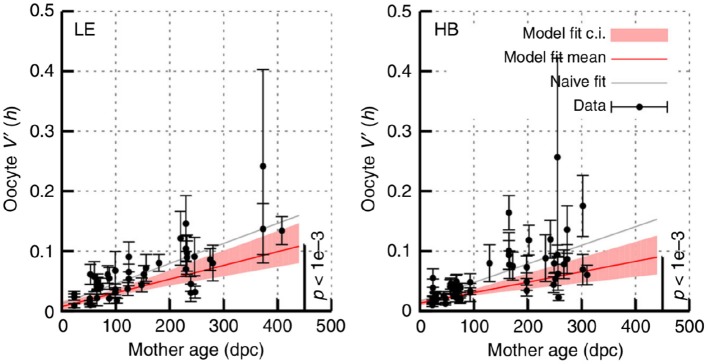
Increasing mutant proportion spread in oocytes with mouse age. Data from Burgstaller et al. (2018), reporting *sample var′* [*h*] in sets of individual oocytes from mice of different ages. HB and LE label two genetic models, involving admixtures of wild-derived haplotypes HB and LE, respectively with haplotype C57Bl/6N. Error bars are derived using Equation (10); “model fit” accounts for this uncertainty and “naive fit” simply fits the bare observations. In all cases a significant linear increase in *sample var′* [*h*] with time, following Equation (11), is observed.

Recent large-scale intergenerational data in mice has shown an ongoing increase in mutant proportion spread in oocytes over time in adult mice (Figure 7). This increasing oocyte-to-oocyte spread of mutant proportion with age has been directly observed in mouse oocytes (Burgstaller et al., 2018), and has been shown to be more statistically supported than a constant-spread model in independent observations in flies, mice, and humans (Johnston and Jones, 2016).

The mechanism(s) behind the ongoing shrinking of the genetic bottleneck (increasing mutant proportion spread) remains unclear (Johnston et al., 2015; Zhang et al., 2018). However, random turnover of mtDNA may be a reasonable candidate mechanism (Johnston et al., 2015; Johnston and Jones, 2016; Burgstaller et al., 2018). The cumulative action of stochastic replication (and degradation) is to generate cell-to-cell spread in mitochondrial statistics, including in mutant proportion. Other processes like diversifying selection, physical clustering, and even mutagenesis could all contribute to the observed increase in spread.

These results are from systems involving cellular admixtures of two main haplotypes. Other results suggest a consistent picture, for example, showing an increasing number of heteroplasmic sites in children from older mothers, which the authors suggest is likely attributable to oocyte aging (Rebolledo-Jaramillo et al., 2014). Another study found non-uniform changes in heteroplasmy with age in humans (Sondheimer et al., 2011).

In light of this observation, this article would advocate an additional careful analysis of the contribution of maternal age to observed mutant proportion patterns. As we expect the genetic bottleneck (mutant proportion spread) to decrease with age, any systematic differences in age between these compared variances could confound other relationships. Conversely and more positively, appropriate accounting for age would help increase the statistical power of these comparisons.

## 6. The Problem of Selection

Throughout the above, we have alluded to the problems that systematic selection for one or more mtDNA types can cause in these analyses. Theory describing the influence of selection has been established, but is complicated (Johnston et al., 2015). In particular, if approaches that assume the absence of selection are used when it is in fact present, errors can arise in estimates of genetic properties and physical mechanisms. As pointed out above, these issues may lead to dramatic underestimation of “bottleneck size,” and cannot be assumed to “cancel out.”

Several of the results above are valid only in the absence of selection: when no mtDNA type experiences an advantage over any other. This is known to be false for many mtDNA pairings in many somatic tissues, where selection for one mtDNA type over another is often observed (reviewed in Burgstaller et al., 2014). Selection in the germline has been more debated, but evidence is increasing. In several studies, the transmission of pathological mutations seems to be subject to selective pressure. The maximum level of transmission for the 3243 mutation in humans has appeared to be limited (Monnot et al., 2011; Otten et al., 2018), and selection against severe mtDNA mutations has been observed in mice (Fan et al., 2008). Recent observations in mice (Burgstaller et al., 2018; Latorre-Pellicer et al., 2019) and humans (Wei et al., 2019) have indeed observed selection at different loci. Burgstaller et al. (2018) suggest that selection may act in different directions at different developmental stages (very recently supported by Latorre-Pellicer et al., 2019), and that these directions may either cancel out or provide a net selective shift. Mathematical theory for the behavior of mutant proportion spread when selection is present remains less well-developed and represents an important future theoretical target. The birth-death-partitioning approach in references (Johnston et al., 2015; Johnston and Jones, 2016) can account for selection but are mathematically complicated. Otten et al. (2018) have proposed a truncated Kimura distribution to describe a selective regime where mutant proportions above a certain value are prohibited, and found that it is supported by observations of the 3243 mutation.

Comparison to the Kimura distribution is often used to argue for an absence of selection. However, this approach must be interpreted with caution. Depending on the mechanism of selection, Kimura-distributed samples may be observed even when selection has occurred. In particular, as approaches using the Kimura distribution sometimes use several “after” but no “before” measurements, it is possible that an early shift in mutant proportion will not be detected.

## 7. Conclusions

### 7.1. The Variable “Genetic Bottleneck”

This article has attempted to review the various models and mechanisms that have been considered for the “genetic bottleneck” (mutant proportion spread). Some diversity in reported mtDNA behavior comes from the choice of analysis protocol: the use of bottleneck parameters, rather than bottleneck sizes that allow a choice of “generation number,” can help avoid this. The reporting of *sample var′* [*h*], the fundamental observation from which these statistics are derived, and its associated uncertainty, will also help interpretability and comparison.

Ongoing research has provided evidence that the “genetic bottleneck” (mutant proportion spread) varies with age, species, individual, and genetic features. Intriguingly, the coupling of physical and genetic behavior of mitochondria (Equation 11; Tam et al., 2013; Aryaman et al., 2019) suggests that heterogeneity in mitochondrial dynamics may induce heterogeneity in mutant proportion.

A diverse range of studies on the mtDNA bottleneck continues to provide a wealth of insight into this important process. However, the very diversity of this research risks confusion arising, particularly around aspects of the prevailing terminology. This article has attempted to clarify some of the concepts involved, to serve as a reference for the increasingly interdisciplinary community working in this field.

Some takehome messages for reference include:

- The “genetic bottleneck” is a readout of mutant proportion spread that is generally not an observable physical quantity, and is measured reported in diverse ways through the literature;
- Observations of mutant proportion spread can have substantial uncertainty both from sampling and technical error, particularly if under 10 samples are used (when the standard error can approach half the observation);
- The physical mechanisms underlying the “genetic bottleneck” (mutant proportion spread) include a combination of copy number reduction (a physical bottleneck), random replication and degradation of mtDNA molecules, and random partitioning at cell divisions;
- The magnitude of the physical bottleneck appears to be flexible, as flexibility in mtDNA turnover can compensate to produce the same effects on mutant proportion spread;
- The presence of mtDNA selection complicates estimates of mutant proportion spread, and different experimental designs report different statistics in this case;
- The “genetic bottleneck” (mutant proportion spread) likely varies by species, individual, age, and mtDNA sequence.

## Data Availability Statement

This study did not generate new experimental data. Code for the simulations and visualizations involved is publically available at https://github.com/StochasticBiology/bottleneck-review.

## Author Contributions

The author confirms being the sole contributor of this work and has approved it for publication.

### Conflict of Interest

The author declares that the research was conducted in the absence of any commercial or financial relationships that could be construed as a potential conflict of interest.

## Footnotes

^1^Other mechanisms, outside the scope of this article, exist to mitigate mtDNA mutation in other taxa (Johnston and Burgstaller, 2019).
